# Pathological complete response of hepatocellular carcinoma confirmed by conversion hepatectomy following atezolizumab plus bevacizumab therapy: a case report and literature review

**DOI:** 10.1007/s12328-023-01895-7

**Published:** 2023-12-10

**Authors:** Shun Sato, Taku Aoki, Takatsugu Matsumoto, Takayuki Shiraki, Shozo Mori, Yukihiro Iso, Takehiko Nemoto, Toshihiko Onishi, Makoto Iijima, Kazuyuki Ishida

**Affiliations:** 1 Department of Hepato-Biliary-Pancreatic Surgery, Dokkyo Medical University, 880 Kitakobayashi, Mibu, Tochigi 321-0293 Japan; 2 Department of Gastroenterology, Dokkyo Medical University, Mibu, Tochigi Japan; 3 Department of Diagnostic Pathology, Dokkyo Medical University, Mibu, Tochigi Japan

**Keywords:** Hepatocellular carcinoma, Atezolizumab, Bevacizumab, Conversion surgery, Complete response

## Abstract

The combination regimen of atezolizumab plus bevacizumab (Atezo/Bev) is currently used as first-line treatment in patients with unresectable hepatocellular carcinoma. Herein, we report a rare case of curative hepatic resection performed as conversion surgery in a patient with intermediate-stage hepatocellular carcinoma following preoperative Atezo/Bev therapy. After five treatment cycles of Atezo/Bev therapy, followed by four cycles of atezolizumab monotherapy, the tumor marker levels decreased to baseline levels and 22 small daughter nodules disappeared, leaving only the primary tumor. Therefore, we performed resection of the primary tumor as conversion surgery, and postoperative histopathology confirmed complete tumor necrosis. No cancer recurrence has been observed until the 5-month postoperative follow-up, and the patient remains drug free. Consistent with the findings in this case, a review of previously reported cases revealed that in cases of successful conversion surgery, neoadjuvant Atezo/Bev therapy was associated with intra-tumoral bleeding, immune-related adverse events, and normalization of the tumor marker levels.

## Introduction

Hepatocellular carcinoma (HCC) is one of the most commonly diagnosed malignancies and the sixth leading cause of cancer-related death worldwide [1]. The Barcelona Clinic Liver Cancer (BCLC) staging system has been used for staging HCCs, and intermediate-stage HCCs have been further subdivided into three stages since the 2022 revision [2]. This development was mainly ascribed to the introduction of new systemic chemotherapies for this cancer. It has been reported that in a subset of patients with intermediate-stage HCC in whom transarterial chemoembolization (TACE) was the only available treatment option, improved prognosis has been achieved with newer chemotherapy regimens [3, 4].

The IMbrave150 trial showed that as compared with sorafenib, combined atezolizumab (an anti-programmed death ligand 1 (PD-L1) monoclonal antibody) plus bevacizumab (an anti-vascular endothelial growth factor (VEGF) monoclonal antibody) (atezo/bev) therapy yielded significantly prolonged overall survival and progression-free survival in patients with unresectable HCC [3, 5, 6]. At present, atezo/bev is established as the first-line systemic therapy for patients with advanced HCC and some patients with intermediate-stage HCC; it also represents the first immunotherapy regimen developed for HCC.

That group in the intermediate stage consists of patients with diffuse, infiltrative, and extensive hepatocellular lesions.

Recently, there have been a few reports of curative conversion surgery after atezo/bev therapy in patients with HCC [7–10]. However, it is not yet known how the process of conversion therapy can be followed. Herein, we report a rare case of pathological complete response (CR) confirmed by conversion hepatectomy after neoadjuvant atezo/bev therapy.

Case presentation.

A 67-year-old man diagnosed as having a liver tumor was referred to our hospital for further management. His past medical history included hypertension, hyperlipidemia, gall bladder stone, gastro-esophageal reflux disease, and untreated liver cirrhosis secondary to hepatitis C virus infection. He was on regular treatment with amlodipine, valsartan, a statin, and a proton pump inhibitor. He had smoked 20 cigarettes daily for 48 years, but never drank. There was nothing of note in his family medical history.

His presented with a several months’ history of loss of appetite, and he reported a body weight loss of about 3 kg over the previous 6 months. Physical examination revealed no abnormalities. Laboratory examination of the serum tumor marker levels revealed a serum alpha fetoprotein (AFP) level of 16.8 ng/mL (normal range < 10 ng/mL) and serum des-gamma-carboxyprothrombin (DCP) level of 326 mAU/mL (normal range < 40 mAU/mL), respectively. Liver function was preserved with an albumin–bilirubin (ALBI) score of − 2.87 and the patient was classified into modified ALBI grade I. Tests for hepatitis viral markers showed negative results for hepatitis B surface antigen and hepatitis B virus DNA, but positive result for hepatitis B core antibody; results for both hepatitis C virus antibody and hepatitis C RNA were positive.

**Fig. 1:**
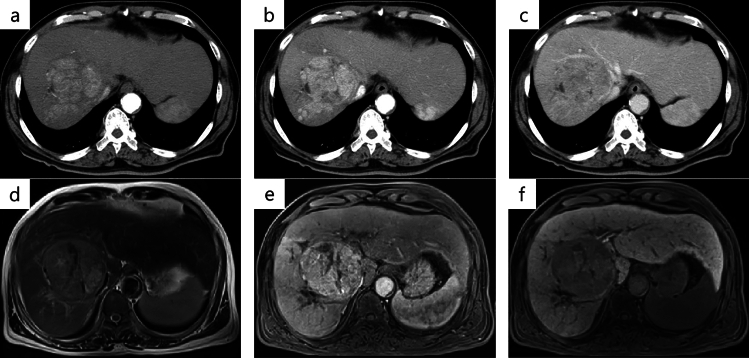
CT and MRI at first visit. **a** Arterial phase of dynamic CT. **b** Portal phase of dynamic CT. **c** Venous phase of dynamic CT. **d** T2-weighted image of MRI. **e** Arterial phase of EOB-MRI. **f** Hepatocyte phase of EOB-MRI. On dynamic CT, the tumor is early contrasted in the arterial phase and washed out in the venous phase. MRI shows a mildly high signal on T2-weighted images. In EOB-MRI, an early contrast effect and a low signal in the hepatocellular phase compared to surrounding normal hepatocytes

Dynamic computed tomography (CT) and magnetic resonance imaging (MRI) were performed. Dynamic CT showed early staining in the arterial phase and washout in the venous phase of the main tumor in segment 8 (Fig. 1). MRI showed a mildly high signal on T2-weighted images, and gadolinium ethoxybenzyl-diethylene-triaminepentaacetic acid enhanced MRI (EOB-MRI) showed that the main tumor has an early contrast effect in the arterial phase and a low signal in the hepatic cell phase compared to the surrounding liver tissue (Fig. 1). Based on these imaging studies, we diagnosed hepatocellular carcinoma.

**Fig. 2:**
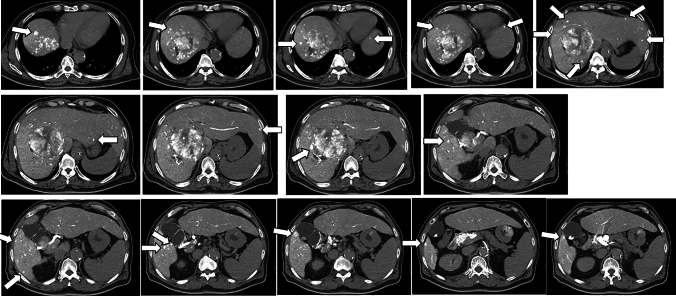
CTHA showed multiple daughter tumors within the liver. Twenty-two contrast-enhanced nodules are present in both lobes (white arrow).

We performed computed tomography during hepatic angiography (CTHA) and computed tomography during arterial portography (CTAP), and detected a liver tumor in segment 8, measuring 81 mm in maximum diameter. In CTAP, the right branch of portal vein failed to be visualized due to compression by the tumor. In addition, CTHA showed 22 smaller daughter tumors, both around the main tumor and in the left hepatic lobe (Fig. 2). According to the BCLC staging classification, we diagnosed the patient as having intermediate-stage HCC. There are multiple tumors in both lobes, the patient was judged as being unsuitable for TACE. Therefore, we started the patient on systemic therapy with atezolizumab 1200 mg plus bevacizumab 924 mg every 3 weeks.

**Fig. 3:**
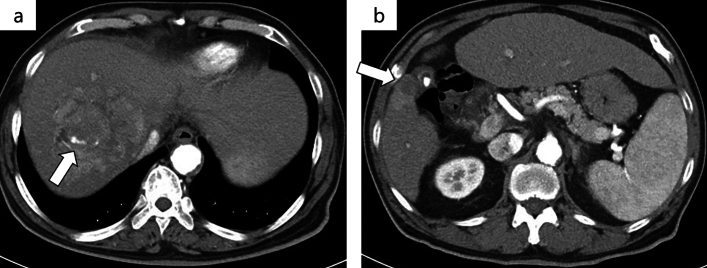
CT after 3 courses of atezo/bev therapy. **a** The main tumor had intra-tumoral hemorrhage (white arrow). **b** The other daughter tumors were not contrasted and tended to necrosis (white arrow)

**Fig. 4:**
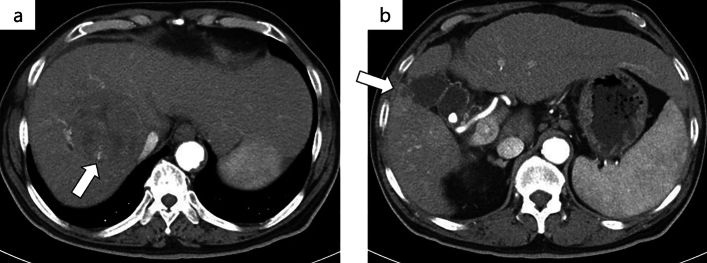
**a** The main tumor had intra-tumoral hemorrhage as before. **b** The other daughter tumors were not contrasted, tended to necrosis, and had shrunk

**Fig. 5:**
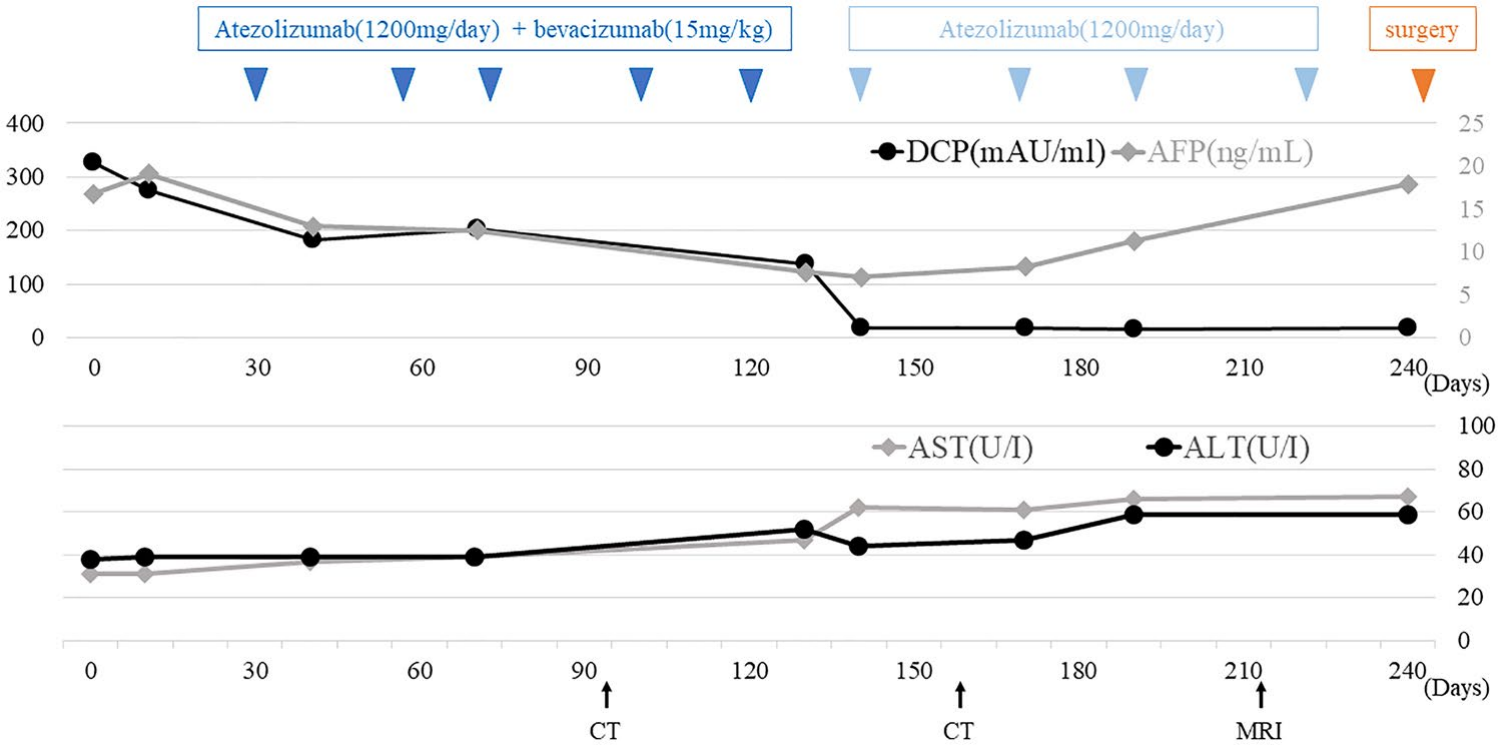
Clinical course during atezo/bev. AFP, which had once tended to decline, gradually increased, so imaging tests were performed again, but the tumor was shrinking, so surgery was performed. Retrospectively, AST and ALT also began to rise around the same time

**Fig. 6:**
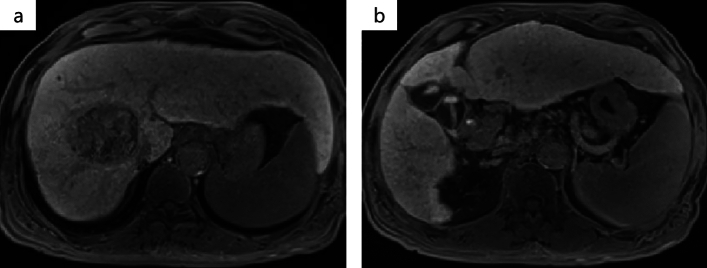
EOB-MRI after 5 courses of Atezo/bev and 3 times atezolizumab monotherapy. **a** In the hepatocyte phase, the main tumor continued to shrink. **b** In the hepatocyte phase, other lesions showed no lower signal than the surrounding normal liver tissue

**Fig. 7:**
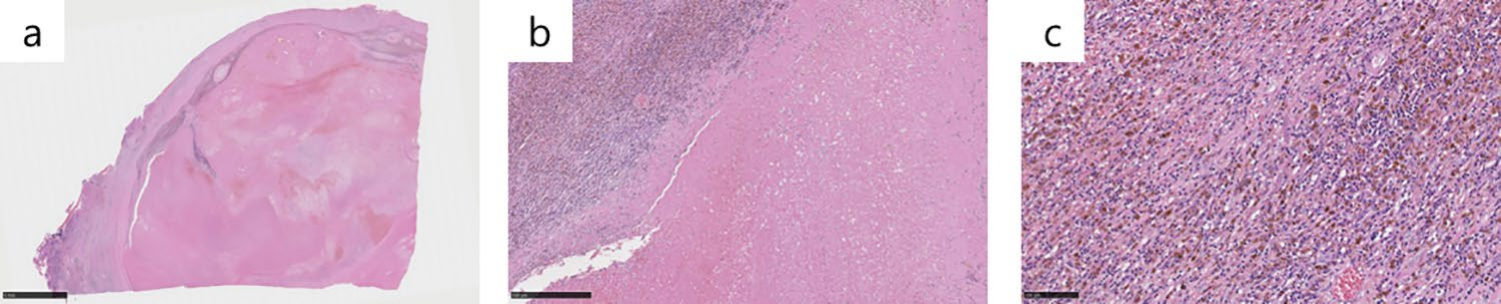
Histological findings of the resected liver specimen. **a** Grossly, a 6*5*4 cm tumor with a fibrous capsule is present, in which the interior part is occupied by necrosis and hemorrhage, and a fibrous septum is seen (hematoxylin and eosin staining, × 1) **b** Inflammatory cell infiltration and hemorrhagic necrosis are shown inside the fibrous connective tissue septa (hematoxylin and eosin staining, × 60) **c** The inflammatory cells, predominantly lymphocytes, are admixed with hemosiderin deposits (hematoxylin and eosin staining, × 200). The presence of lymphocytic infiltration may not necessarily represent an atezo/bev-specific effect, since microhemorrhages naturally occur when tumor cells collapse in response to treatment, which induce chronic inflammatory cell infiltration, including lymphocytes

After three cycles of atezo/bev treatment, a monitoring CT revealed that the largest tumor had grown slightly from 81 to 87 mm, with no change in the number of small tumors; however, some of the small tumors showed no contrast enhancement and were considered as having necrotized. In addition, there was leakage of contrast medium within the largest tumor, suggestive of intra-tumoral bleeding **(**Fig. 3**)**. The tumor response to atezo/bev therapy was assessed as Stable Disease (SD) based on RECIST 1.1 (Response Evaluation Criteria in Solid Tumors, version 1.1), and we continued the same treatment. After 5 cycles, bevacizumab was discontinued as the patient developed proteinuria, and atezolizumab alone was continued (atezolizumab monotherapy). Repeat CT performed after five cycles of atezo/bev therapy and one cycle of atezolizumab monotherapy showed that the small daughter nodules had disappeared, and that the diameter of the largest tumor had decreased from 81 to 65 mm. The intra-tumoral bleeding persisted (Fig. 4). Laboratory examination at this time revealed normalization of the tumor marker levels. We continued the patient on atezolizumab monotherapy; however, the serum AFP level began to rise gradually again, and we repeated the imaging studies again (Fig. 5). No new lesions were detected, and the maximum diameter of the main tumor had decreased further to 54 mm (Fig. 6). The tumor had shrunk, while the marker levels had begun to rise again, so that we decided to perform conversion surgery while the tumor was still resectable: eight months after the start of atezo/bev treatment, we performed anterior segmentectomy of the liver. The tumor was completely resected, and postoperative histopathological examination revealed that all the cancer cells were necrotic and that there were no viable HCC cells in the tumor (Fig. 7). Furthermore, there were hemosiderin deposits and inflammatory cell infiltration, mainly consisting of lymphocytes, within the tumor. The patient developed postoperative complications, including Clavien–Dindo grade IIIa bile leakage and Clavien–Dindo grade I ascites. However, 5 months have elapsed since the surgery, and follow-up examinations have revealed no evidence of recurrence of the intrahepatic lesions or appearance of any distant metastases, and the patient remains drug free.

## Discussion

In the present patient diagnosed as having intermediate-stage HCC, pathological complete response was confirmed by conversion surgery after neoadjuvant atezo/bev therapy. In the IMbrave 150 trial, the OS rate in the atezo/bev arm was significantly higher than that in the sorafenib arm. In addition, atezo/bev therapy was associated with a high overall response rate (ORR) of 44% in patients with intermediate-stage HCC and 27% in patients with advanced HCC as assessed according to RECIST 1.1, suggesting a tumor shrinkage effect of the treatment [5, 6].

Based on the results of this trial, Kudo argued that if atezo/bev is effective, it is important to consider conversion to curative therapy, such as resection or TACE, rather than just continuing atezo/bev therapy^4)^. However, there have not been many case reports of conversion surgeries. [7–16].

The clinical CR rate in the atezo/bev group in the IMbrave 150 trial was 8%. However, 47.9% of patients in this trial had previously received local tumor treatments such as TACE. Kudo noted that pathological CR with systemic therapy alone is rare [4].

Cases of conversion surgery for HCC reported previously and our own case are shown in Table 1. Based on a review of these cases, we would like to discuss the merits and means of transition to conversion surgery.

**Table1:** Previous reports of conversion hepatectomy after atezo/bev

References	Age, Sex	Etiology	Initial treatment before atezo/bev	HCC at start of atezo/bev						Atezo/bev cycles	IrAE	Intra-tumoral hemorrhage	Tumor-marker normalization	Resected tumor	Outcome (cancer-free/drug-free)
				BCLC grade	Size maximum	Vascular invasion or compression	Metastasis	AFP (ng/mL)	DCP (mAU/mL)						
[7]	77 M	Alcohol	None	C	16.8	None	Lung	1.4	43,124	7	Hepatitis	+	+ (DCP)	Necrosis	+ / +
[8]	63 M	ND	TACE	B	ND	None	None	ND	ND	15	ND	ND	ND	Viable	+ / +
[9]	77 M	HBV	TACE, HAIC	C	15	Hepatic vein	Adrenal	759.0	5681	9	ND	ND	+ (AFP, DCP)	Necrosis	+ / +
[10]	79 M	ND	TACE	C	ND	Portal vein	None	3.2	20	2	Skin disorder	ND	+ (stable before treatment)	Viable	+ / +
[11]	74 M	ND	None	C	ND	Hepatic vein (HVTT)	None	52.0	11,676	3	ND	ND	+ (AFP, DCP)	Necrosis	+ / +
[12]-1	76 M	ND	None	A	13	Hepatic & Portal vein	None	ND	1202	8	ND	ND	+ (DCP)	Slight residual viable	+ / +
[12]-2	72 M	ND	HAIC	C	ND	Portal vein (PVTT)	None	11.0	4	5	ND	ND	+ (AFP)	necrosis	+ / +
[13]-1	71 M	Alcohol	None	A	12	Portal vein	None	106.6	67,200	10	ND	ND	+ (AFP, DCP)	necrosis	+ / +
[13]-2	72 M	None	None	A	12	Portal vein	None	570.6	65,143	7	ND	ND	+ (AFP, DCP)	Slight residual viable	+ / +
[14]	75 M	ND	TAE (due to rapture at first)	C	5.7	None	Peritoneal	8.9	2,163	15	Neuropathy	ND	+ (DCP)	Necrosis	+ / +
[15]	30 M	None	None	C	9	None	Lymph node	9.8	ND	4	Colitis	ND	+ (AFP)	viable	+ / +
[16]	60 M	HBV	None	C	ND	Portal vein (PVTT)	None	14,696.0	2,141	1 (1 atezo monotherapy followed)	None	ND	+ (AFP)	Necrosis	+ / +
Our case	67 M	HCV	None	B	8.1	Portal vein	None	16.8	326	5 (4 atezo monotherapy followed)	None	+	+ (DCP)	Necrosis	+ / +

*HCC* hepatocellular carcinoma, *atezo/bev* atezolizumab and bevacizumab combination therapy, *BCLC* grade Barcelona Clinic Liver Cancer grade, *HBV* hepatitis B virus, *TACE* transcatheter arterial chemoembolization, *HAIC* hepatic arterial infusion chemotherapy, *HVTT* hepatic vein tumor thrombosis, *PVTT* portal vein tumor thrombosis, *AFP* alpha fetoprotein, *DCP* des-γ-carboxy prothrombin, *irAE* immune-related Adverse Event, *ND* no data

Conversion therapy in patients with HCC offers the following advantages: (i) potential to achieve cancer-free and drug-free status; (ii) the possibility of evaluation of the pathologic response to chemotherapy; and (iii) the possibility of making future treatment choices, in the event of disease recurrence, based on the results of immunohistochemical analysis or cancer genome profiling. In our case, in terms of the treatment efficacy, liver resection performed as conversion surgery may have been unnecessary, because our postoperative histopathology revealed that complete tumor necrosis had already been achieved with the neoadjuvant atezo/bev therapy alone. Nevertheless, this effect of atezo/bev therapy would have been difficult to evaluate without surgery, as the tumor had still not disappeared on imaging.

A review of the reported cases of conversion surgery in the literature suggests that about 7 courses of atezo/bev, on average and median, were administered prior to surgery (Table 1). In our patient reported herein, we repeated the imaging during the atezo/bev therapy when the serum AFP level began to increase gradually, and found that fortunately, the tumor had not only shrunk in size, but was also resectable. Therefore, we performed conversion surgery. In retrospect, however, in our patient, even the CT performed after five cycles of atezo/bev therapy and one course of atezolizumab monotherapy showed that the tumor had become resectable, and we could have scheduled conversion surgery at that time point rather than continue atezolizumab monotherapy.

The optimal interval to conversion surgery after bevacizumab withdrawal, which has the side effect of delayed wound healing, remains controversial. Previous reports have recommended withdrawal of bevacizumab at least 5 to 8 weeks prior to the surgery, and resumption of the drug not earlier than 28 days after surgery or only after confirming complete wound healing [17–19].

Of the **eight** reported cases in which pathological CR was confirmed by conversion surgery, two showed leakage of contrast medium into the tumor, suggestive of intra-tumoral bleeding, during the course of atezo/bev therapy. It was not mentioned in the other 6 cases. Intra-tumoral bleeding causes tumor necrosis by inducing hypoxia, and a previous report has suggested that intra-tumoral hemorrhage is associated with a favorable prognosis in cases of HCC [20].

In addition, four of the previously reported cases of successful conversion surgery after atezo/bev treatment developed immune-related adverse events (irAEs) [7, 21, 22]. There are no reports of a statistical association between the development of irAEs and the efficacy of treatment against HCC yet, but such an association has been reported for other cancer types treated with immune checkpoint inhibitors (ICIs) [23, 24]. There are reports of existence of an association between the development of irAEs and treatment efficacy based on histopathological findings, but not in cases of HCC [25, 26].

Retrospectively, in our case, a very mild irAE may have occurred. AFP was gradually elevated, so surgery was performed, but the tumor was necrosis; when the reason for the elevated AFP was examined, AST and ALT were slightly increased by atezolizumab, as shown in Fig. 5. AST, ALT, and AFP normalized after postoperative withdrawal of the drug. In other words, the increase in AFP does not reflect the tumor but may have been re-elevated by chemotherapy-induced hepatitis. To determine if this hepatitis was irAE, the noncancerous areas of the resected liver were re-examined. The hepatitis was accompanied by a high degree of lymphocytic infiltration. This finding was not indicative of the presence of irAE, due in part to cirrhosis caused by hepatitis C (Fig. 7). Recent reports have demonstrated that PD-L1 is also expressed in normal hepatocytes and that atezolizumab causes hepatocellular necroptosis in human hepatocyte lines [27].

In addition, normalization of the serum levels of the tumor markers AFP and DCP was observed following atezo/bev therapy in all cases, except one that was not mentioned. Reduction of the tumor marker levels are indicative of a favorable tumor response, but normalization of the tumor marker levels is needed to achieve conversion surgery successfully.

In summary, in all the reported cases of successful conversion surgery, including ours, about **seven** courses of atezo/bev were administered until the patients were transitioned to conversion surgery. In cases of successful conversion surgery, irAE and normalization of serum tumor marker values often developed during the neoadjuvant atezo/bev treatment period, and intra-tumoral hemorrhage was observed in some cases although analysis of data from further cases is needed to confirm our findings.

## Abbreviations

AFP: Alpha fetoprotein
ALBI score: Albumin–bilirubin score
Atezo/Bev: Combination regimen of atezolizumab plus bevacizumab
BCLC stage: The Barcelona Clinic Liver Cancer stage
CR: Complete response
CTAP: Computed tomography during arterial portography
CTHA: Computed tomography during hepatic angiography
DCP: Des-gamma-carboxyprothrombin
HCC: Hepatocellular carcinoma
ICI: Immune checkpoint inhibitor
irAEs: Immune-related adverse events
PD-L1: Programmed death ligand 1
RECIST 1.1: Response Evaluation Criteria in Solid Tumors, version 1.1
TACE: Transarterial chemoembolization
VEGF: Vascular endothelial growth factor
